# Down-regulated Solute Carrier Family 4 Member 4 Predicts Poor Progression in Colorectal Cancer

**DOI:** 10.7150/jca.36696

**Published:** 2020-03-26

**Authors:** Hong Yang, Yao Lu, Weilan Lan, Bin Huang, Jiumao Lin

**Affiliations:** 1 Academy of Integrative Medicine, Fujian University of Traditional Chinese Medicine, Fuzhou, Fujian 350122, P.R. China; 2 Fujian Key Laboratory of Integrative Medicine on Geriatrics, Fujian University of Traditional Chinese Medicine, Fuzhou, Fujian 350122, P.R. China

**Keywords:** SLC4A4, colorectal cancer, bioinformatical analysis, differentially expressed genes, survival

## Abstract

**Aim**: To identify potential key candidate genes, whose expression and clinical significance was further assessed in colorectal cancer (CRC).

**Methods**: Three original microarray datasets (GSE41328, GSE22598, and GSE23878) from NCBI-GEO were used to analyze differentially expressed genes (DEGs) in CRC. Online database analyses through Oncomine and GEIPA were performed to evaluate SLC4A4 expression and explore the prognostic merit of SLC4A4 expression, which was further confirmed by analyses from QPCR based cDNA array and IHC based tissue microarray (TMA). STRING website was used to explore the interaction between SLC4A4 with other DEGs based on the protein-protein interaction (PPI) networks.

**Results**: Analysis of three original microarray datasets from GEO identified 82 shared, differentially expressed genes (28 upregulated and 54 down-regulated) in CRC tissues. Online analyses from Oncomine and GEIPA revealed lower SLC4A4 mRNA expression in CRC tissues compared to adjacent normal tissues, which were further confirmed by QPCR based cDNA array and IHC based TMA analyses on both mRNA and protein levels. Survival analyses through GEIPA and from TMA demonstrated that low SLC4A4 expression is correlated with worse overall survival among patients with CRC. Survival analysis from Kaplan-meier plotter demonstrated that low SLC4A4 expression is significantly associated with poor progression (including relapse-free survival, overall survival, distant metastasis-free survival, post-progression survival) of patients with breast cancer, lung cancer, gastric cancer, and ovarian cancer. PPI analysis found that SLC4A4 is highly correlated with various genes, including SLC9A3, SLC26A6, ENSG00000214921, SLC26A4, SLC9A3R1, and SLC9A1.

**Conclusion**: The mRNA and protein levels of SLC4A4 were decreased in CRC tissues, and low expression of SLC4A4 significantly correlated with shorter survival of CRC patients and poorer progression of patients with breast cancer, lung cancer, gastric cancer and ovarian cancer, suggesting potential role of SLC4A4 on tumor suppression and prognostic prediction in multiple malignancies including CRC.

## Introduction

Colorectal cancer (CRC) is the third most common malignancy and the fourth leading cause of cancer-related death in the world, resulting in more than half a million deaths every year^1^-^3^. In the United States, in 2017, CRC was ranked third in frequency of incidence (135,430 new cases of CRC were diagnosed, with 40.7 cases per 100,000 individuals); further, CRC is more common in men than in women^4^. Although decreased tobacco consumption and red meat intake and increased aspirin use have contributed disproportionately to declining CRC morbidity and mortality, this disease is still highly lethal (14.8 deaths per 100,000 individuals)^5^,^6^. Because of the high morbidity and mortality associated with CRC, the identification of more valuable and convenient biomarkers for early diagnosis and survival prediction is critically important and in high demand.

Recent studies in the field of molecular pathology have introduced thousands of tumor biomarkers associated with the progression or prognosis of different types of cancers. Many of these biomarkers have been evaluated for patient survival in CRC, such as TP53, KRAS and CDKN1A^7^-^9^. Gene chip analysis is a gene-detection technique that has been in use for >10 years; this technique can detect all the genes within the same sample time-point expression information and is particularly suitable for screening differentially expressed genes^10^. The Gene Expression Omnibus (GEOs) is a repository of gene expression data, wherein a large number of microarray data has been deposited and stored^11^. A considerable amount of microarray data has been accumulated on CRC^12^, and hundreds of differentially expressed genes (DEGs) have been identified. The innovative combination of integrated bioinformatics methods with expression profiling techniques may allow the identification of reliable biomarkers for CRC.

In this study, we downloaded three original microarray datasets (GSE41328^13^, GSE22598^14^ and GSE23878^15^) from the NCBI-Gene Expression Omnibus database (NCBI-GEO) (available online: https://www.ncbi.nlm.nih.gov/geo), from which data of total 62 CRC cases and 51 normal colon mucosa is available. Then, we filtered DEGs in the GEO2R tool using conventional data processing standards. Combination of online database (including Oncomine, GEIPA and Kaplan-meier plotter), Q-PCR based cDNA array and IHC based TMA were further performed to assess the expression of selected DEGs and its correlation with survival patients of CRC, as well as progression (including RFS, Relapse Free Survival; OS, Overall Survival; DMFS, Distant Metastasis Free Survival; PPS, Post Progression Survival) of patients with breast cancer, lung cancer, gastric cancer or ovarian cancer. Finally, using Search Tool for the Retrieval of Interacting Genes (STRING) (available online: http://string-db.org/), the present study further explores the interaction between SLC4A4 with other DEGs based on protein-protein interaction (PPI) networks^16^, ^17^.

## Materials and Methods

### Microarray data information and DEGs identification

NCBI-GEO is a free database of microarray/gene profiles and next-generation sequencing, from which we obtained the CRC and normal GSE22598, GSE23878, and GSE41328 datasets. All the datasets were based on Affymetrix HG-U133 Plus 2.0 arrays. The microarray data in GSE22598 included 17 pairs of cancer and non-cancerous tissues from CRC patients (Submission date: December 04, 2011) 13. The microarray data in GSE23878 was based on 35 CRC tissue samples and 24 non-cancerous colorectal tissue samples (Submission date: August 30, 2011)^14^. Lastly, the microarray data in GSE41328 included 10 colorectal adenocarcinoma tissues and matched normal colonic tissues (Submission date: October 03, 2012)^15^.

GEO2R was employed to detect DEGs between CRC tissues and noncancerous colorectal tissues 11. Differentially expressed genes (DEGs) were identified using the classical *t*-test, and statistically significant DEGs were defined with *P* < 0.01 and [logFC] > 2 as the cutoff criteria. The co‑expressed upregulated and downregulated DEGs in the two gene expression profiles were identified with a Venn Diagram (available online: http://bioinformatics.psb.ugent.be/webtools/Venn/).

### Candidate gene SLC4A4 mRNA expression analysis thorough Oncomine

The overview of SLC4A4 expression in various tumors specimens and its detailed expression in CRC specimens (24 datasets), as well as in noncancerous normal controls were analyzed by Oncomine platform (Available online: https://www.oncomine.org)^18^. The folds change of SLC4A4 expression in different datasets ware listed in Table 2. Details of standardized normalization techniques and statistical calculations are provided on the Oncomine website.

### SLC4A4 mRNA expression and survival analyses through GEIPA

The mRNA expression of SLC4A4 in CRC specimens was further confirmed by GEPIA^19^. GEPIA is a web-based tool to deliver fast and customizable functionalities based on TCGA and GTEx data^19^. We utilized the COAD-TCGA and READ-TCGA in GEPIA to analyze the expression of SLC4A4 in CRC and its correlation with overall survival of CRC patients.

### SLC4A4 mRNA expression analysis by QPCR based tissue cDNA array

A CRC cDNA microarray was purchased from Shanghai Outdo Biotech Company (Shanghai, China), which contains 30 paired cancer and non-cancerous colorectal tissues. We performed Q-PCR analyses using an ABI 7500 Fast Real-Time PCR System (Applied Biosystems, Carlsbad, CA, USA). Then, 1 μL cDNA was used for PCR amplification with SLC4A4-specific primers. GAPDH was used as the internal standard to control, and the mRNA level was assessed using a threshold cycle value, for which the formula is 2-ΔΔCT, where ΔCT = [CT (target gene) - CT (GAPDH)].

### IHC based TMA and survival analyses

TMA containing CRC (n=90) and paired noncancerous colorectal tissues (n=90) were purchased from Shanghai Outdo Biotech Company (Shanghai, China). All 90 specimens used for microarray were obtained from Taizhou Hospital of Zhejiang Province with reliable information on survival (no censorship prior to 7 years of follow-up), which was approved by the Ethics Committee of Taizhou Hospital of Zhejiang Province, in accordance with the principles of Declaration of Helsinki. The protein expression of SLC4A4 in CRC samples was determined by IHC. Briefly, the section was incubated with antibody against SLC4A4 (1:3000; Cat No. 32777; Signalway Antibody LLC, Maryland, MD, USA) using the standard technique^20^. The stained slide was scanned with Nano Zoomer 2.0 HT slide scanner (Hamamatsu Photonics, Hamamatsu, Japan). The intensity (no staining, 0; weak, 1; moderate, 2; strong, 3) and percentage (1-25% positive, 1; 26%-50% positive, 2; 51%-75% positive, 3; 76%-100% positive, 4) of positively stained cells were analyzed by experienced pathologists blinded to the clinical and pathological data. The expression of SLC4A4 in CRC samples was assessed by IHC score, which was calculated by the following formula: final score= intensity score × percentage score. To further explore the correlation between SLC4A4 protein expression in CRC tissues with overall survival of CRC patients, the expression of SLC4A4 in CRC tissues was divided into two categories: high expression (IHC score: 6-12) and low expression (IHC score: 0-5). The correlation was analyzed by Kaplan-Meier estimates and compared using log-rank test.

### Correlation analysis between SLC4A4 expression and survival of patients with malignancies though Kaplan-meier plotter

Kaplan-Meier Plotter (www.kmplot.com) is an online database, containing survival information (including OS, RFS, PPS, DMPS, FP or PFS) of patients with breast cancer, lung cancer, gastric cancer or ovarian cancer. The expression of SLC4A4 in malignancies patient samples were divided into two groups by median expression (high vs. low expression), and the correlation between SLC4A4 mRNA expression with survival of patients was analyzed by a Kaplan-Meier survival plot using log rank test.

### Integration of the PPI network of SLC4A4

It is believed that the complex interactions between molecules, such as protein-protein interactions (PPI), play a vital role in organismal and cellular biochemistry. Therefore, we further analyzed the interaction between DEGs based on PPI networks^21^, and construct a PPI network by integrating the proteins interacted with SLC4A4 using the STRING website^16^, ^17^, ^22^.

### Statistical analysis

The Pair-samples t-test was performed to compare the gene expression in tumor tissues and adjacent normal tissues in cDNA array and TMA. All statistical tests were performed using SPSS 19.0 (Chicago, IL, USA), *P*-value of <0.05 was considered statistically significant.

## Results

### Identification of differentially expressed genes in CRC

**Figure 1:**
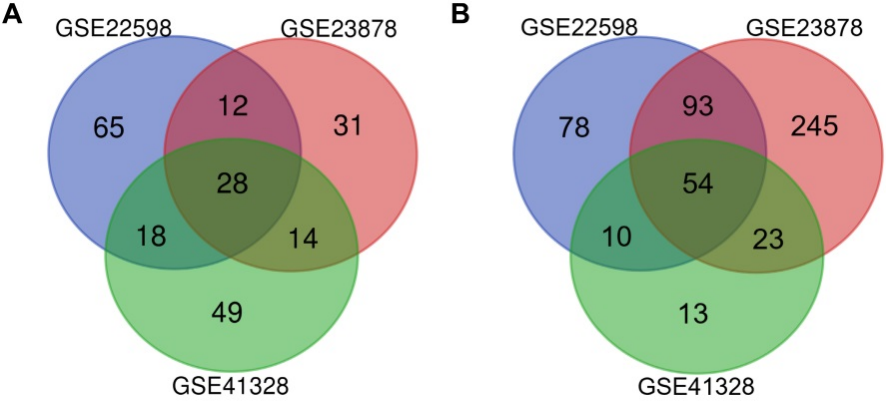
** Identification of differentially expressed genes in CRC.** Draw Venn Diagram (http://bioinformatics.psb.ugent.be/webtools/Venn/) was used to identify DEGs among three cohort profile datasets (GSE22598, GSE23878, and GSE41328). The 28 upregulated genes **(A)** and 54 downregulated genes **(B)** in the CRC datasets were showed. The DEGs were identified with the classical t-test, and statistically significant DEGs were defined with *P* < 0.01 and [logFC] > 2 as the cutoff criteria.

NCBI-GEO is a free database of microarray/gene profiles and next-generation sequencing, from which CRC and normal or adjacent mucosa tissue gene expression profiles of GSE22598, GSE41328, and GSE23878 were obtained. Using *P <* 0.01 and [logFC] > 2 as the cutoff criteria, after integrated bioinformatics analysis, a total of 82 consistently expressed genes were identified from the three profile datasets, including 28 upregulated genes and 54 downregulated genes in the CRC tissues compared with the normal colon tissues (Fig. 1 and Table 1).

### Low mRNA and protein expression of SLC4A4 in CRC

**Figure 2:**
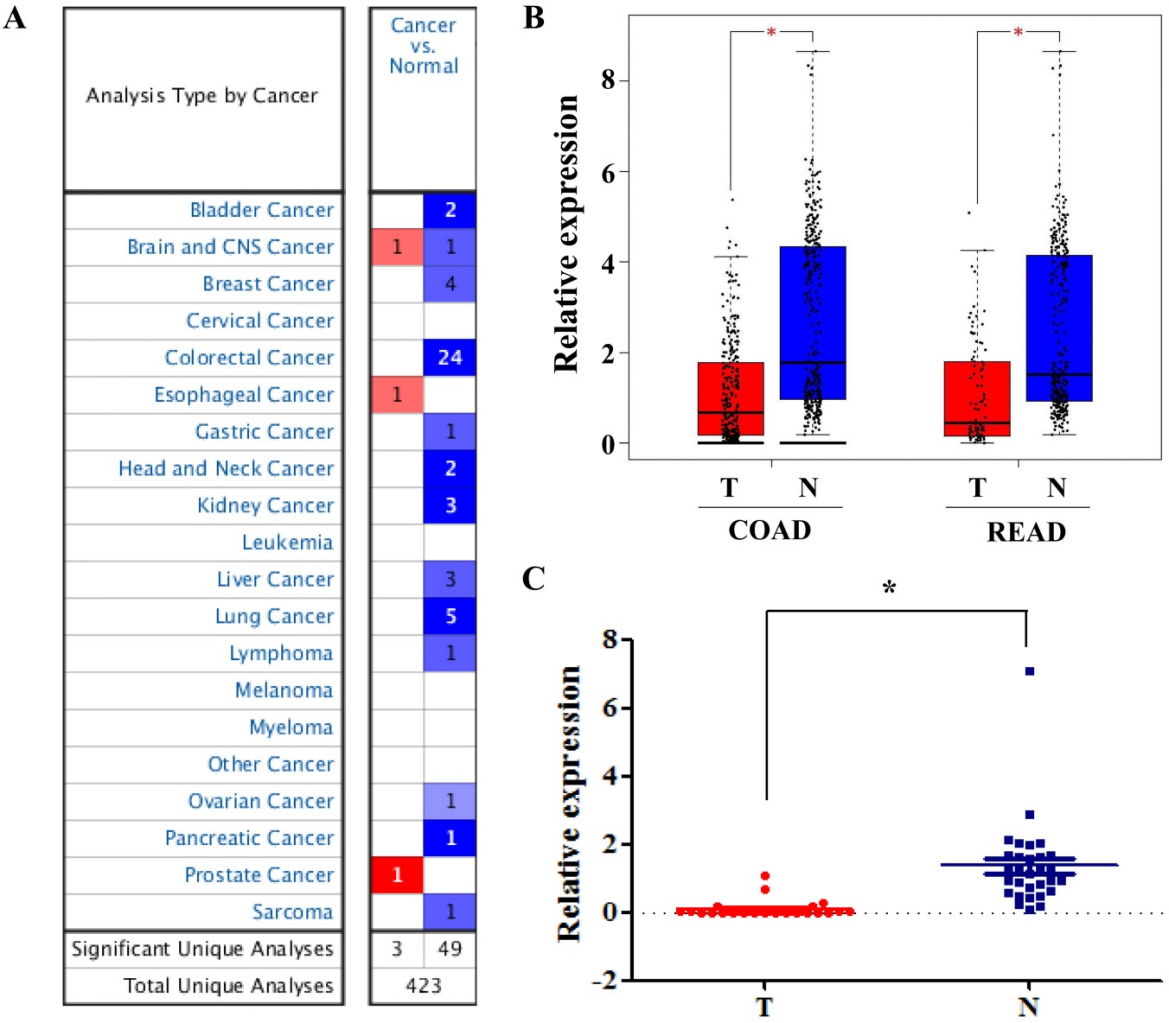
** The mRNA expression of SLC4A4 in CRC. (A)** The overview of SLC4A4 mRNA expression in various tumors was analyzed through Oncomine. **(B)** The mRNA expression of SLC4A4 was analyzed using TCGA-COAD and TCGA-READ datasets through GEIPA. **(C)** SLC4A4 mRNA expression in 30 pairs of CRC tissues and noncancerous colorectal tissues (cDNA array) was measured by QPCR analysis. T: tumor tissues; N: normal tissues. *P*<0.05, vs. noncancerous colorectal tissues.

**Figure 3:**
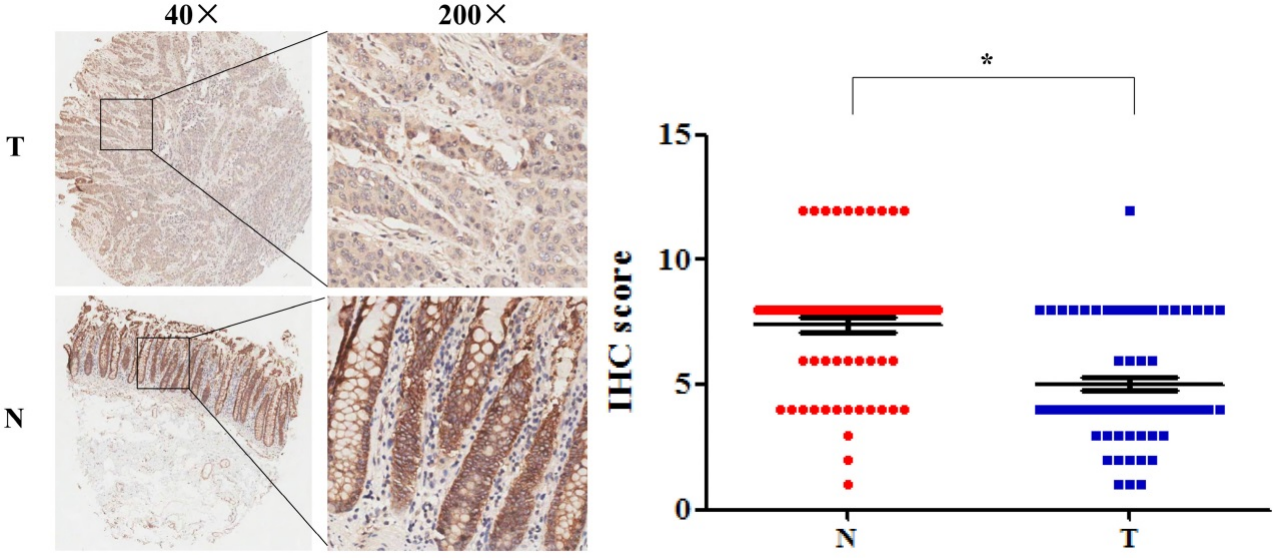
** The protein expression of SLC4A4 in CRC.** SLC4A4 protein expression in 90 pairs of CRC tissues and noncancerous colorectal tissues were analyzed using an IHC-based tissue microarray (TMA). Representative images were taken at a magnification of 40× or 200× (left panel). The IHC score was showed in right panel. T: tumor tissues; N: normal tissues. *P<0.05, vs. noncancerous colorectal tissues.

Among these DEGs, we found that SLC4A4 is one of the down-regulated genes in CRC tissues (Table 1), while its role in CRC remained largely unknown, and therefore was selected for the further study. The overview of SLC4A4 expression through Oncomine demonstrated that the SLC4A4 mRNA expression in most datasets from various types of tumors was obviously decreased (Fig. 2A). Notably, SLC4A4 mRNA expression was significantly down-regulated in CRC tissues of 24 datasets from Oncomine database (Fig. 2A and Table 2), which consistent with the analysis from both TCGA-COAD and TCGA-READ datasets through GEIPA (Fig. 2B; P<0.05). The decrease of SLC4A4 mRNA expression in CRC tissues was further confirmed by analyses of Q-PCR based cDNA array (Fig. 2C; P<0.05) and IHC based TMA (Fig. 3; P<0.05), indicating that the expression of SLC4A4 in CRC tissues were down-regulated comparing with noncancerous colorectal tissues.

### Lower SLC4A4 expression associated with shorter survival of CRC patients

**Figure 4:**
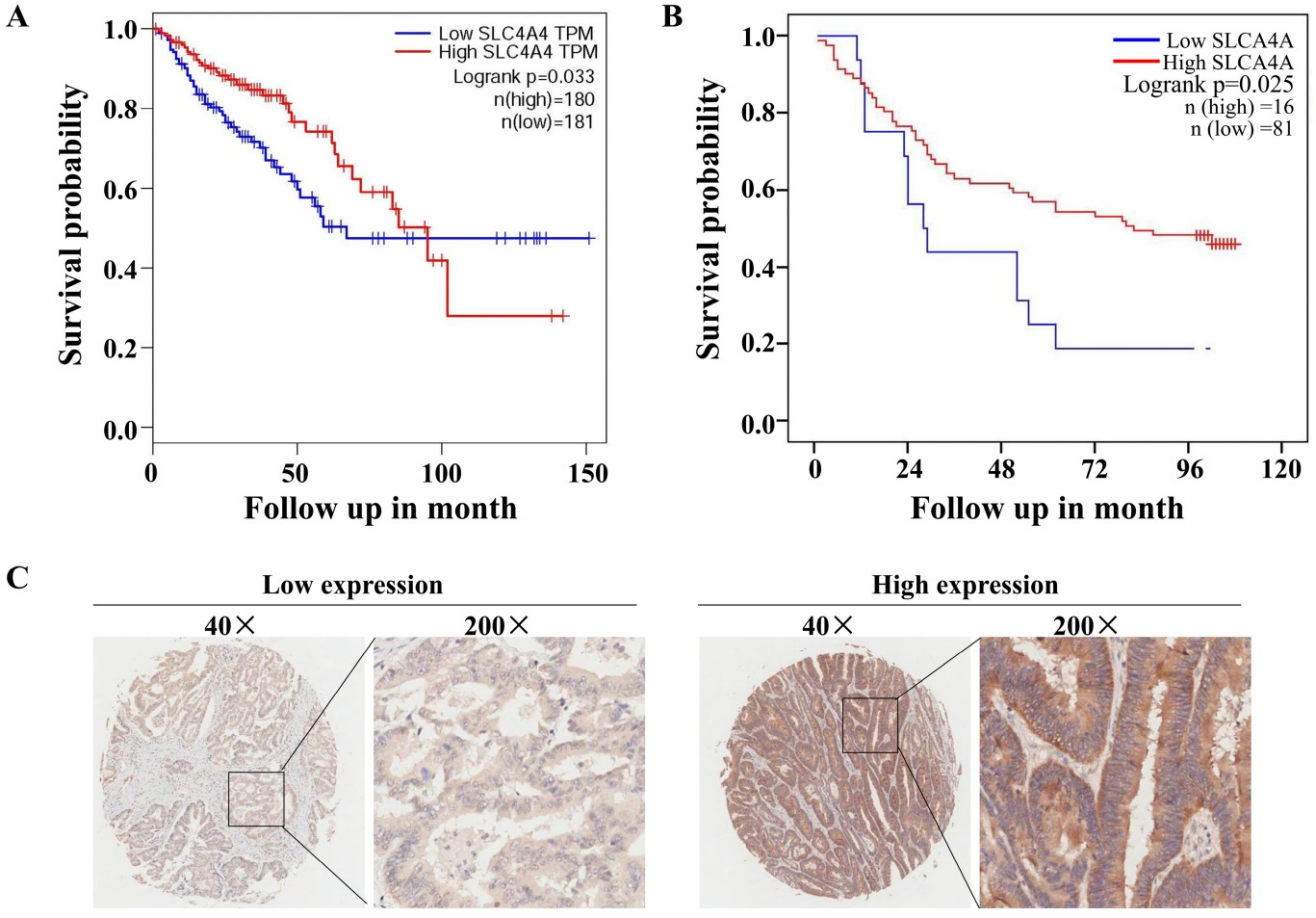
** Correlation between SLC4A4 expression and overall survival of CRC patients. (A)** The correlation between SLC4A4 mRNA expression and overall survival (OS) in patients with CRC was analyzed using log-rank tests based on SLC4A4 expression in CRC tissues from the database of TCGA through GEIPA website. **(B)** The association between SLC4A4 protein expressions with overall survival of CRC patients was analyzed based on SLC4A4 expression in CRC tissues. **(C)** Representative images of high or low PNO1 expression in CRC tissues were taken at a magnification of 40× or 200×.

The correlation analysis using TCGA database through GEIPA website revealed that low levels of SLC4A4 mRNA expression in CRC tissues were significantly correlated with worse overall survival (OS) (P<0.05) among patients with CRC (Fig. 4A). Based on the protein expression of SLC4A4 in CRC tissues of TMA, survival analysis indicated that lower SLC4A4 protein expression obviously associated with shorter survival of CRC patients (Fig. 4B; P<0.05), the representative image of high or low protein expression of SLC4A4 were shown in Fig. 4C. Association analyses between SLC4A4 expression and clinical characteristics didn't found obviously correlation between SLC4A4 expression with Age, Gender, Pathology, Tumor size, T stage, N stage and M stage (Table 3). These studies indicated that lower SLC4A4 expression correlated with shorter overall survival of CRC patients and suggest a potential role of lower SLC4A4 expression on prediction of poor prognosis.

### Lower SLC4A4 expression indicates poorer progression of patients with malignancies

**Figure 5:**
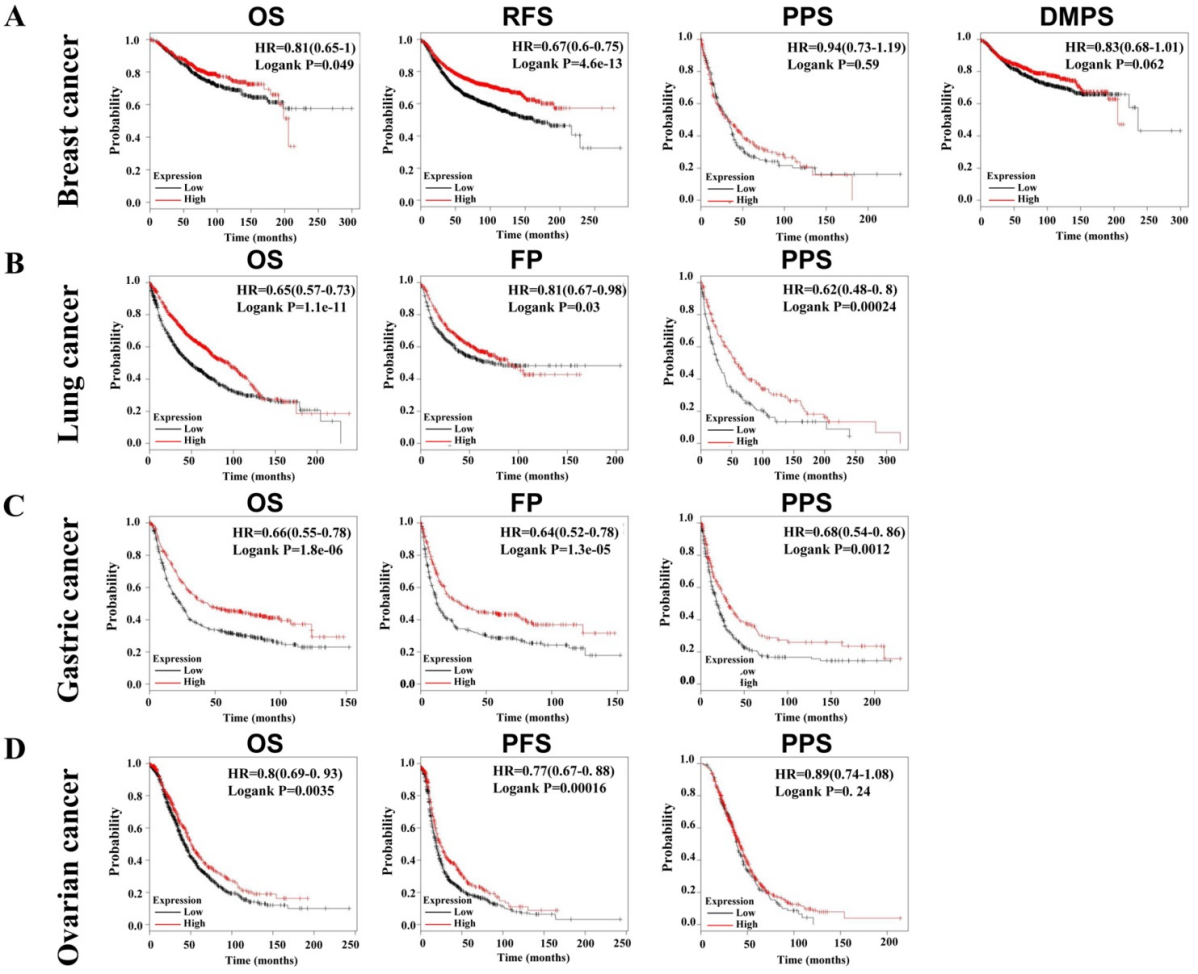
** Correlation between SLC4A4 expression and survival of patients with malignancies.** The correlation of SLC4A4 mRNA expression with OS, RFS, PPS, DMPS of breast cancer patients **(A)**, OS, FP and PPS of lung cancer patients **(B)** and gastric cancer patients** (C)**, as well as OS, PFS and PPS of ovarian cancer patients** (D)** were analyzed using log-rank tests through Kaplan-meier plotter.

Due to the decrease of SLC4A4 mRNA expression in multiple malignancies (Fig. 2A), including breast cancer, lung cancer, gastric cancer, and ovarian cancer, we further assessed the correlation between SLC4A4 mRNA expression and survival of patients, including OS, RFS, PPS, DMPS, FP or PFS. As showed in Fig. 5, lower expression of SLC4A4 obviously correlated with OS, RFS, PPS, DMPS of patients with breast cancer (Fig. 5A, P<0.05), OS, FP and PPS of patients with lung cancer (Fig. 5B, P<0.05) and gastric cancer (Fig. 5C, P<0.05), as well as OS, PFS and PPS of patients with ovarian cancer (Fig. 5D, P<0.05). These analyses revealed that lower SLC4A4 significantly associated with poorer progression of patients with malignancies, including breast cancer, lung cancer, gastric cancer, and ovarian cancer.

### Network analysis of SLC4A4 with interacted genes

**Figure 6:**
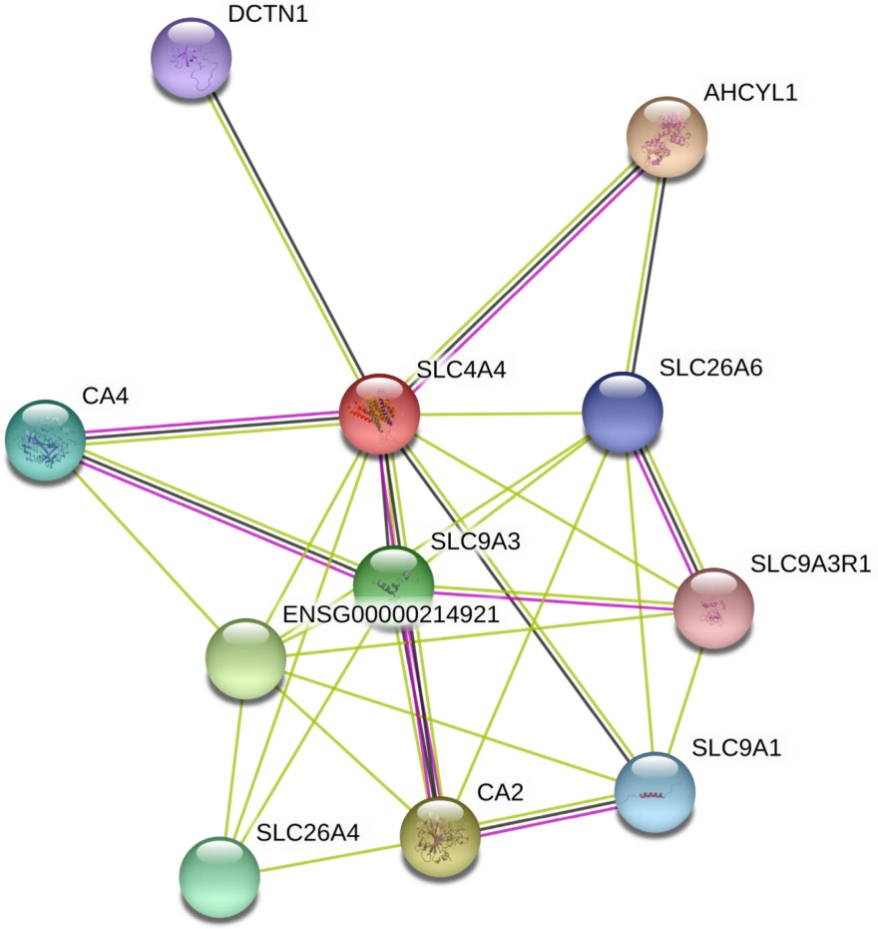
** Analysis of SLC4A4 related proteins based on PPI network.** Using the STRING database, a total of 11 proteins were filtered into the proteins PPI network.

The interaction network among DEGs was plotted using the STRING database for protein interaction based on Protein-Protein Interaction (PPI) network. As shown in Fig. 6, PPI analysis showed known and predicted interactions interaction between SLC4A4 with SLC9A3, SLC26A6, ENSG00000214921, SLC26A4, DCTN1, AHCYL1, CA4, SLC9A3R1, SLC9A1, and CA2, most of which had been reported be oncogenes or tumor suppressors in cancers (including CRC)^23^-^26^.

## Discussion

CRC is the third most commonly diagnosed cancer and the third leading cause of cancer-related death in men and women in the United States^6^, ^27^. Many basic and clinical studies have aimed to reveal the causes and underlying mechanisms of CRC development and progression over the past few decades, and despite significant improvements in the diagnosis, treatment, and survival prediction of CRC, novel biomarkers for prognosis are yet to be identified^28^. To further identify oncogene or tumor suppressor, we analyzed the profile datasets of three cohorts from research groups using bioinformatics methods, revealing 28 upregulated and 54 downregulated genes within the 82 consistently expressed genes. Due to the significantly decrease of SLC4A4 levels in CRC tissues and its role in CRC remained largely unknown in most of tumors including CRC, and therefore were selected as a candidate tumor suppressor gene.

SLC4A4, also known as electrogenic sodium bicarbonate cotransporter isoform 1 (NBCe1), is widely expressed in the secretory and absorptive epithelia and plays an important role in intracellular pH regulation and transepithelial HCO_3_^-^ transportation^29^. Basolaterally located SLC4A4 is responsible for HCO_3_^-^ reabsorption in the proximal tubule of the kidneys^30^. Furthermore, SLC4A4 is responsible for basolateral HCO_3_^-^ uptake prior to luminal secretion in the epithelial lining of the pancreatic duct^31^ and the small intestine^32^. Glial NBCe1 operates with a stoichiometry of 1 Na^+^: 2 HCO_3_^-^ and can transport Na^+^ and bicarbonate in both directions across the glial cell membrane^33^-^35^. Na^+^-coupled acid-base transporters play essential roles in human biology, and their dysfunction has been linked to cancer, as well as heart and brain disease^36^. The dysregulation of Na^+^-coupled acid-base SLC transporters in cancer cells has important diagnostic and therapeutic implications^37^. The study of SLC4A4 in colon cancer cells revealed that knockdown of SLC4A4 via shRNA reduced cell proliferation and mortality during external acidosis and spheroid growth, while pHi recovery from acidosis was partially reduced with knockdown of SLC4A4^38^, while overexpression of SLC4A4 obviously suppressed the proliferation and metastasis of Caki-1 cells^39^, demonstrating that the role of SLC4A4 in cancers including CRC urgent need to be further addressed.

To further assess the expression of SLC4A4 in CRC tissues and its correlation with survival of CRC patients. In the current study, database analysis from Oncomine and GEIPA further confirmed the down-regulation of SLC4A4 mRNA expression in CRC tissues when compared to noncancerous colorectal tissues, which is consistent with the result of QPCR-based cDNA array analysis. Moreover, IHC based TMA analysis revealed the decrease of SLC4A4 protein expression in CRC tissues. These studies indicated that both mRNA and protein expression of SLC4A4 were significantly down-regulated in CRC tissues, which might play an essential role during the development of CRC. In addition, we also used the GEIPA website and IHC based TMA analysis to explore the prognostic merit of SLC4A4 expression. Our results showed that low levels of SLC4A4 expression were significantly correlated with shorter OS in CRC patients. Unfortunately, we didn't find the correlation between SLC4A4 expression with the Age, Gender, Pathology, Tumor size, T stage, N stage, M stage of CRC patients, it might due to the limit of sample number in the current study. These studies suggest that a decrease of SLC4A4 might be a common event in CRC and be a valuable predictor for prognosis of CRC patients. Survival analysis based on SLC4A4 expression in multiple malignancies (including breast cancer, lung cancer, gastric cancer, and ovarian cancer) demonstrated that lower SLC4A4 expression significantly correlated with poorer progression (including OS, RFS, PPS, DMPS, FP or PFS) of patients. These studies suggested the potential value of lower SLC4A4 expression on prognostic prediction.

To further predict the potential function of SLC4A4 in CRC, using the PPI network, we analyzed the interaction among 82 abnormal expressed genes and found that SLC4A4 might interact with SLC9A3, SLC26A6, ENSG00000214921, SLC26A4, DCTN1, AHCYL1, CA4, SLC9A3R1, SLC9A1, and CA2. Some of these known or predicted interacted genes had been reported to be oncogenes or tumor suppressor genes 23-26. For example, SLC9A3 an exchanger of Na^+^/H^+^, regulates the transepithelial absorption of Na+ and water and is principally expressed in the apical membranes of the intestinal epithelium, renal proximal tubule, epididymis, and vas deferens 40. Loss of the SLC9A3 allele may result in diminished Na^+^ and HCO3^-^ absorption, which suggests that SLC9A3 plays a vital role on the apical membranes of the intestinal epithelium, renal proximal tubule, epididymis, and vas deferens 34. However, the biological function and underlying mechanism of SLC4A4 in CRC should be further addressed in our further nearly studies.

In summary, using profile datasets from multiple cohorts and integrated bioinformatics analysis, we identified the decrease of SLC4A4 in CRC tissues, which was confirmed on both mRNA and protein levels. Moreover, low SLC4A4 expression significantly associated with shorter overall survival of CRC patients. Prediction of interaction between DEGs suggested that by interaction with SLC9A3 and other genes might be one of the mechanisms of SLC4A4 participated during the development of CRC.

## Figures and Tables

**Table 1:** 82 differentially expressed genes (DEGs) were identified from three profile datasets, including 28 up-regulated genes and 54 down-regulated genes in the colorectal cancer tissues, compared to normal tissues.

DEGs	Genes Name
Up-regulated	CDH3, SLCO4A1, LY6G6F, COL11A1, NFE2L3, CEMIP, AZGP1, MMP7, CTHRC1, TRIB3, TACSTD2, FOXQ1, MMP3, TESC, KLK10, CLDN2, CXCL5, ASCL2, INHBA, AJUBA, MMP1, SLC7A5, CXCL8, CRNDE, CLDN1, EPHX4, KRT23, DPEP1
Down-regulated	LGALS2, NR3C2, SPIB, HSD17B2, ABCG2, GUCA2B, CHP2, SCARA5, CLCA4, DHRS11, AKR1B10, ARL14, CA4, TRPM6, NXPE4, PYY, SCIN, B3GALT5, TSPAN7, CA2, FCGBP, PKIB, ANPEP, CEACAM7, PADI2, ADTRP, KLF4, ABCA8, SLC51B, ADH1B, GCG, GBA3, MS4A12, PCK1, CPM, VSIG2, ADH1C, GCNT2, LRRC19, SST, SCNN1B, C2orf88, HPGD, LAMA1, CWH43, BEST4, CA1, MUC4, SLC4A4, CA12, GUCA2A, UGT1A3, DHRS9, CA7

**Table 2:** Oncomine Analysis of SLC4A4 mRNA Expression in Colorectal Cancer.

Cohort no.	Cohort	Data type	Sample(n)	Fold change	P value
1	Hong Colorectal	mRNA	Colorectal Carcinoma (70) vs Normal (12)	-76.322	4.85E-43
2	Kaiser Colon	mRNA	Rectosigmoid Adenocarcinoma (10) vs Normal (5)	-24.78	7.11E-09
		mRNA	Rectal Adenocarcinoma (8) vs Normal (5)	-27.235	1.47E-08
		mRNA	Colon Mucinous Adenocarcinoma (13) vs Normal (5)	-20.693	2.18E-09
		mRNA	Cecum Adenocarcinoma (17) vs Normal (5)	-24.032	6.09E-12
		mRNA	Colon Adenocarcinoma (41) vs Normal (5)	-21.563	6.90E-14
		mRNA	Rectal Mucinous Adenocarcinoma (4) vs Normal (5)	-2.159	5.34E-07
3	Skrzypczak Colorectal	mRNA	Colon Adenocarcinoma (45) vs Normal (24)	-9.951	2.75E-20
		mRNA	Colorectal Carcinoma (36) vs Normal (24)	-9.342	1.80E-23
4	Skrzypczak2 Colorectal	mRNA	Colorectal Carcinoma (5) vs Normal (10)	-20.289	1.07E-09
		mRNA	Colon Adenoma (5) vs Normal (10)	-13.821	3.90E-09
		mRNA	Colon Adenoma Epithelia (5) vs Normal (10)	-16.29	8.16E-11
		mRNA	Colon Carcinoma Epithelia (5) vs Normal (10)	-17.805	7.70E-10
5	Sabates-Bellver Colon	mRNA	Rectal Adenoma (7) vs Normal (32)	-8.076	1.88E-05
		mRNA	Colon Adenoma (25) vs Normal (32)	-6.33	5.65E-20
6	TCGA Colorectal	mRNA	Cecum Adenocarcinoma (22) vs Normal (22)	-24.228	9.79E-16
		mRNA	Rectal Adenocarcinoma (60) vs Normal (22)	-26.086	2.09E-34
		mRNA	Colon Adenocarcinoma (101) vs Normal (22)	-20.712	1.79E-38
		mRNA	Colon Mucinous Adenocarcinoma (22) vs Normal (22)	-14.978	6.34E-13
		mRNA	Rectal Mucinous Adenocarcinoma (6) vs Normal (22)	-11.405	4.13E-06
		mRNA	Rectosigmoid Adenocarcinoma (22) vs Normal (3)	-40.431	3.79E-06
7	Gaedcke Colorectal	mRNA	Rectal Adenocarcinoma (65) vs Normal (65)	-3.71	3.89E-49
8	Ki Colon	mRNA	Colon Adenocarcinoma (50) vs Normal (28)	-4.786	3.87E-14
9	Caspar Colon	mRNA	Colon Adenoma Epithelia (56) vs Normal (22)	-2.957	3.06E-06

**Table 3:** Correlation between SLCA4A expression and clinicopathological characteristics

	Total (N=97)	SLCA4A protein expression		P-value
		Low expression (n=16)	High expression (n=81)	
**Age**				
≤ 65	43 (44.3%)	4 (25.0%)	39 (48.1%)	0.090
> 65	54 (55.7%)	12 (72.0%)	42 (41.9%)	
**Gender**				
Male	53 (54.6%)	11 (68.8%)	42 (41.9%)	0.219
Female	44 (45.4%)	5 (31.2%)	39 (48.1%)	
**Pathology stage**				
Ⅰ	0 (0%)	0 (0%)	0 (0%)	0.751
Ⅱ	51 (52.6%)	9 (56.3%)	42 (41.9%)	
Ⅲ	46 (47.4%)	7 (43.7%)	39 (48.1%)	
**Tumor size**				
≤ 5 cm	50 (51.5%)	10 (62.5%)	40 (49.4%)	0.343
>5 cm	47 (48.5%)	6 (37.5%)	41 (50.6%)	
**T stage**				
T1	1 (1.0%)	0 (0%)	1 (1.2%)	0.852
T2	5 (5.2%)	0 (0%)	5 (6.2%)	
T3	74 (76.3%)	14 (87.5%)	60 (74.1%)	
T4	17 (17.5%)	2 (12.5%)	15 (18.5%)	
**N stage**				
N0	57 (58.8%)	9 (56.3%)	48 (59.3%)	0.872
N1	29 (29.9%)	6 (37.5%)	23 (28.4%)	
N2	11 (11.3%)	1 (6.2%)	10 (12.3%)	
**M stage**				
M0	94 (96.9%)	15 (93.8%)	79 (97.5%)	0.430
M1	3 (3.1%)	1 (6.2%)	2 (2.5%)	

## Abbreviations

SLC4A4: Solute Carrier Family 4 Member 4
CRC: colorectal cancer
DEGs: Differentially expressed genes
QPCR: quantitative-PCR
cDNA: complementary DNA
IHC: immunohistonchemistry
TMA: Tissue microarray
PPI: the protein-protein interaction
GEOs: Gene Expression Omnibus
STRING: Search Tool for the Retrieval of Interacting Genes
RFS: relapse-free survival
OS: overall survival
DMFS: distant metastasis-free survival
PPS: post-progression survival
NBCe1: electrogenic sodium bicarbonate cotransporter isoform 1
